# Improving physical activity in COPD: towards a new paradigm

**DOI:** 10.1186/1465-9921-14-115

**Published:** 2013-10-30

**Authors:** Thierry Troosters, Thys van der Molen, Michael Polkey, Roberto A Rabinovich, Ioannis Vogiatzis, Idelle Weisman, Karoly Kulich

**Affiliations:** 1 Pulmonary Rehabilitation and Respiratory Division, UZ Gasthuisberg, Herestraat 49, B3000 Leuven, Belgium; 2 Department of Rehabilitation Sciences, KU Leuven, Leuven, Belgium; 3 University of Groningen University Medical Center, Groningen, Netherlands; 4 NIHR Respiratory Biomedical Research Unit, Royal Brompton Hospital, National Heart and Lung Institute (Imperial College), London, UK; 5 ELEGI Colt Laboratory, Centre for Inflammation Research. The Queen’s Medical Research Institute, University of Edinburgh, Scotland, UK; 6 Department of Physical Education & Sport Sciences and 1st Department of Respiratory Medicine, National and Kapodistrian University of Athens, Athens, Greece; 7 Novartis Pharmaceuticals Corporation, East Hanover, NJ, USA; 8 Novartis Pharma AG, Basel, Switzerland

**Keywords:** Physical activity, Bronchodilators, Pulmonary rehabilitation, COPD, Activity monitors

## Abstract

Chronic obstructive pulmonary disease (COPD) is a debilitating disease affecting patients in daily life, both physically and emotionally. Symptoms such as dyspnea and muscle fatigue, lead to exercise intolerance, which, together with behavioral issues, trigger physical inactivity, a key feature of COPD. Physical inactivity is associated with adverse clinical outcomes, including hospitalization and all-cause mortality. Increasing activity levels is crucial for effective management strategies and could lead to improved long-term outcomes. In this review we summarize objective and subjective instruments for evaluating physical activity and focus on interventions such as pulmonary rehabilitation or bronchodilators aimed at increasing activity levels. To date, only limited evidence exists to support the effectiveness of these interventions. We suggest that a multimodal approach comprising pulmonary rehabilitation, pharmacotherapy, and counselling programs aimed at addressing emotional and behavioural aspects of COPD may be an effective way to increase physical activity and improve health status in the long term.

## Introduction

**Figure 1:**
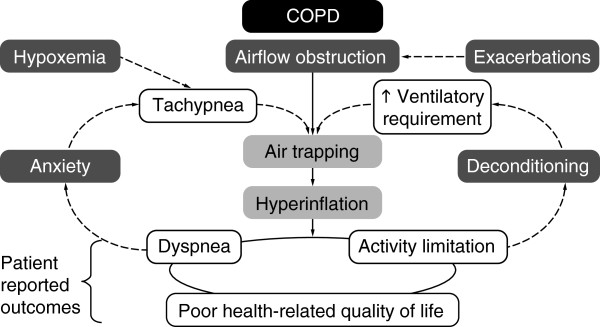
The vicious cycle of inactivity and symptoms.

Chronic obstructive pulmonary disease (COPD) is a debilitating and progressive disease that primarily affects the respiratory system. In many patients, it also has detrimental extra-pulmonary effects, such as weight loss and skeletal muscle dysfunction/wasting [1]. The pulmonary and skeletal muscle abnormalities limit the pulmonary ventilation and enhance the ventilatory requirements during exercise resulting in exercise-associated symptoms such as dyspnea and fatigue. These symptoms make exercise an unpleasant experience, which many patients try to avoid, and along with a depressive mood status (in up to 30% of patients), further accelerates the process, leading to an inactive life-style. Muscle deconditioning, associated with reduced physical activity, contributes to further inactivity and as a result patients get trapped in a vicious cycle of declining physical activity levels and increasing symptoms with exercise (Figure 1) [1-3].

Physical activity levels are remarkably lower in stable outpatients with COPD than in healthy individuals [4-6]; even in patients with early-stages disease [7-9]. At a group level, increasing severity of COPD is associated with decreasing physical activity [9]. Physical activity level is recognized as a predictor of mortality and hospitalization in patients with COPD and contributes to disease progression and poor outcomes [10]. Increasing activity levels may improve long-term outcomes as seen in other chronic conditions such as diabetes [11].

This review will summarize the characteristics of instruments used to assess physical activity in COPD and discuss the important implications of physical inactivity in this context, with a particular focus on interventions aimed at helping patients become more physically active in daily life. The review was based on a literature search of the PubMed database (no date limits) for COPD and terms relating to exercise and physical activity.

### Physical activity levels recommendation and applications in COPD

The recently developed World Health Organization (WHO) guidelines for physical activity recommend that all adults should undertake at least 150 minutes of moderate-intensity aerobic activity per week, such as walking, to maintain a healthy lifestyle [12]. Individuals limited by medical conditions are advised to undertake as much physical activity as their health allows. A joint statement from the American Thoracic Society and the European Respiratory Society in 2006 states that pulmonary rehabilitation 'should no longer be viewed as a “last ditch” effort for patients with severe respiratory impairment. Rather, it should be an integral part of the clinical management of all patients with chronic respiratory disease, addressing their functional and/or psychologic deficits’ [13]. An update of this document, recently accepted for publication, will further stress the importance of physical activity and improvement of physical activity as a goal for pulmonary rehabilitation. The more recent 2013 Global Initiative for Chronic Obstructive Lung Disease (GOLD) strategy recommends that all patients with COPD should participate in daily physical activity, although recommended levels have not been defined [2]. Despite these recommendations, a recent Swedish study demonstrated that significantly fewer patients with COPD attained recommended physical activity levels compared with a healthy population and patients with other chronic diseases, such as rheumatoid arthritis or diabetes [14].

### Evaluation of physical activity

It is important to make the distinction between physical activity and exercise capacity, which are both closely related to clinical outcomes in COPD. Physical activity is 'any bodily movement produced by skeletal muscles that results in energy expenditure’ [15]. By contrast, exercise capacity indicates an individual’s ability to endure exercise, where exercise comprises physical activities that are specifically performed with the intention of improving physical fitness. Exercise capacity indicates what a person is capable of doing, while physical activity reflects what someone actually does.

Physical activity can be assessed by direct observation, evaluation of energy expenditure during bodily movement, physical activity questionnaires and patient diaries, and the use of performance based motion sensors. Direct observation is a time-consuming and intrusive method, and therefore not suitable for assessing physical activity in large populations [16]. Energy spent on physical activities can be assessed by indirect calorimetry such as the doubly labeled water method [17]; however, body mass, movement efficiency, and the energy cost of activities, make inter-individual comparison of the amount of physical activity performed difficult. Furthermore, the quantity, duration, frequency and intensity of physical activity cannot be discriminated. Moreover, patients with COPD have a poor mechanical efficiency yielding larger energy expenditure compared to healthy subjects for the same level of activity [18].

### Subjective instruments for assessing physical activity

Specifically designed questionnaires and diaries are subjective measures that have been used to quantify physical activity in daily life [19,20]. These tools are helpful for evaluating the patients’ perspectives on their ability to carry out daily activities. Self-reported questionnaires and diaries rely on memory and recall of the patients [21,22] and several variables such as the design of the questionnaire [23], patient characteristics (age, cognitive capacity, cultural factors) [16,21,24] and interviewer characteristics [16] may affect the reliability of the results. It has been shown that patients’ estimation of time spent on physical activities in daily life disagreed with objective assessment [25].

The most frequently used subjective tools with a better-documented validation include the Minnesota Leisure Time Physical Activity Questionnaire (MLTPAQ) or Survey (MLTPAS) [26], the Baecke Physical Activity Questionnaire [27,28], Follick’s diary [29], the Physical Activity Scale for the Elderly (PASE) [30,31] and the Zutphen Physical Activity Questionnaire (ZPAQ) [32]. A recent study in which the utility of four questionnaires was compared against accelerometry in COPD found the Stanford to be more reliable than the PASE, Zutphen or Baecke [33]. Unfortunately the association between measured PA and the questionnaires outcomes was poor for all questionnaires. Web-based applications, which require less time than paper-based questionnaires to be completed by patients, have also been developed [34]. A unique project aimed at developing and validating patient reported outcome tools to investigate dimensions of physical activity that are judged as being essential by patients, is currently underway (PROactive; physical activity as a crucial patient reported outcome in COPD) and is due to be completed in 2014 [12]. This project is exploring the development of tools that will capture daily physical activity from the patient perspective to reflect their experiences of physical activity.

### Objective instruments

The clinical evaluation and validation of objective measures of assessing physical activity continues to be investigated. The PROactive project has identified available physical activity monitors [35]. Motion sensors, which include pedometers used for measuring steps and accelerometers used for detecting body acceleration, can be used for the objective quantification of physical activity over time [16]. Although pedometers may underestimate the amount of physical activity, particularly slow-walking [36], and offer no information on the pattern of physical activity or the time spent in different activities [37], several studies have shown that they can capture physical activity in patients with COPD [38-40].

Accelerometers are electronic devices, generally worn on the arm (multisensory armband devices) or waist, which estimate physical activity outcomes such as body posture, quantity and intensity of body movements, energy expenditure, and physical activity level based on measurements of body’s acceleration [41].

Evidence for the reliability, validity and responsiveness of accelerometers is still limited in the COPD population [9,16]. Accelerometers are also limited by the cost, poor patient acceptance of some models [42], sensitivity to artefacts [16], observation bias [43] and compliance issues [43]. However, a recent multicentre study reported good compliance with wearing the devices and limited technical problems [44]. Despite these limitations, two recent studies [45,46] provide a framework to validate activity monitors for use in patients with chronic disease, evaluating compliance, usability, validity in the field setting and in a laboratory setting. Three of six monitors tested met all prespecified validity criteria and can be used to assess physical activity levels of patients with COPD. Physical activity is variable from day to day, which is a challenge to clinical trial designs. More studies elaborating on how physical activity monitor outcomes can be assessed and reported is needed. Guidance is available from a recent series of papers endorsed by the American College of Sports Medicine [47].

### Implications of physical inactivity in COPD

**Figure 2:**
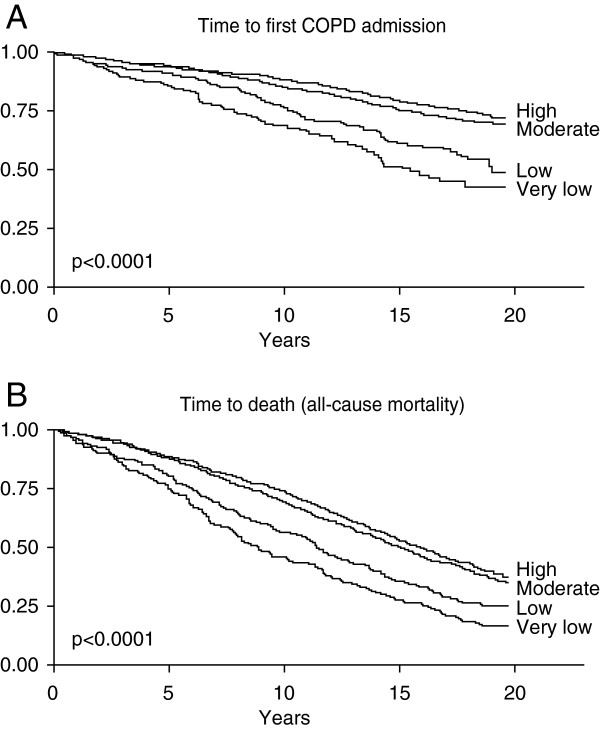
**Regular physical activity reduces hospital admissions (upper panel A) and all-cause death (lower panel B) **[50]**.** Kaplan-Meier curves according to level of regular physical activity during follow-up. Patients with COPD from the Copenhagen City Heart Study (n=2386), recruited from 1981 and followed to 2000. Reproduced from [50] with permission from BMJ Publishing Group Ltd.

Physical inactivity is one of the most potent predictors of mortality in COPD [48,49]. A population-based study found that all levels of regular physical activity were associated with an adjusted lower risk of all-cause mortality and respiratory mortality (Figure 2) [50]. Low levels of physical activity have been associated with a higher risk of hospitalization and re-hospitalization [48,50,51]. In a prospective study in 173 patients with moderate-to-very severe COPD, patients with low physical activity levels (measured objectively) had a shorter time to first COPD admission versus those with higher activity levels [48]. Patients are particularly inactive during and after hospitalization for an acute exacerbation, and physical inactivity soon after discharge has been shown to increase the probability of readmission within the following year [52].

It has been shown that the level of physical activity is the most important factor in determining self-rated general health and HRQoL in patients with COPD, with the most physically active patients reporting the best health and HRQoL [4,53]. Over a five year study period, patients who maintained moderate or high levels of physical activity or increased their physical activity had an improved HRQoL, and patients who had a low level of physical activity experienced significant declines in HRQoL [53]. Further, a cross-sectional, survey-based study with over 1500 patients with COPD reported that the probability of having better self-rated general health increased 2.4–7.7-fold and the likelihood of experiencing psychological distress declined by around 50% with higher physical activity levels [4]. Overall, patients with COPD who are more physically active have generally better functional status in terms of diffusing capacity of the lung carbon monoxide, expiratory muscle strength, exercise capacity, maximal oxygen uptake and systemic inflammation, compared with those who are less active [10].

Considering this relationship between physical activity and meaningful patient outcomes, improving physical activity levels is an important goal in the management of COPD.

### Interventions to improve physical activity

#### *Pulmonary rehabilitation and the modification of patient behavior*

Pulmonary rehabilitation (PR) aims to break the vicious cycle illustrated in Figure 1. PR programs are multidisciplinary programs that are built around an exercise training intervention. Exercise training aims to reverse the systemic consequences of COPD, in particular the skeletal muscle dysfunction, enhances the mechanical efficiency of physical activities (particularly walking) and reduces the sensitivity to dyspnea [54] and the ventilation required to overcome a specific task [55]. PR also appears to have a beneficial effect on patients’ experience of physical activity, e.g. by reducing fear and allowing them to increase activities [56].

PR programs have shown varying results with respect to their effect on physical activity. Three studies, both short-term (3 weeks) and longer-term (6–12 weeks) did not find an increase in the level of physical activity after the PR programs [57-59]. However, three studies reported a significant increase in physical activity after PR for the same duration [60-62]. Issues regarding the measurement properties of the activity monitors, the best site (or sites) to wear the motion detectors, and the optimal variable to analyze (movement intensity/duration, estimated steps, estimated energy expenditure, etc.) may all play a role. Only recently have studies of these motion detector devices in COPD begun to emerge [45,46]. Although exercise training may confer a significant increase in physical activities [63], these improvements are smaller than expected if considering that exercise training can result in a substantial increase in exercise endurance [8].

In healthy subjects, behavioral and environmental factors are associated with physical activity levels (Reviewed in Bauman et al. [64]). It has been hypothesized that the reduced physical activity levels in patients with COPD may potentially have a behavioral element and some patients may opt to limit their activity levels rather than be restricted by their symptoms [65]. In a study with COPD patients, a counseling program that used pedometers to monitor and motivate patients to increase their level of physical activity was offered to a group of out-clinic patients [39]. After 12 weeks, the patients who received counseling showed a significant increase in the number of steps per-day and a significant improvement in arm and leg strength, HRQoL, and changes in intrinsic motivation score to be physically active compared with patients who received usual care. Other studies have shown that counseling programs and pulmonary rehabilitation or structured exercise programs can be complementary [38,66]. More recently internet based programs have found their way to the COPD population [67]. These programs provide feedback to patients and may foster the creation of social networks that invite to be physically active.

### Pharmacotherapy

Dynamic hyperinflation, which is associated with reduced physical activity levels [68,69], is improved with bronchodilator therapy [70], indicating indirectly that bronchodilation could result in increased physical activity. Indeed, this had been suggested by Casaburi et al. in a trial assessing the effect of tiotropium on exercise endurance. Patients receiving tiotropium showed significantly longer exercise endurance time at the conclusion of pulmonary rehabilitation compared to patients in the placebo control group [71]. Notably, a following study suggested that tiotropium could amplify the effectiveness of pulmonary rehabilitation as shown by the increase in patient self-reported participation in physical activities. A subsequent sub-analysis of the Understanding Potential Long-term Impacts on Lung Function with Tiotropium (UPLIFT) trial indicated that therapy with tiotropium was effective in improving the QoL of patients with COPD, particularly with regard to the SGRQ domain of physical activity [72].

**Figure 3:**
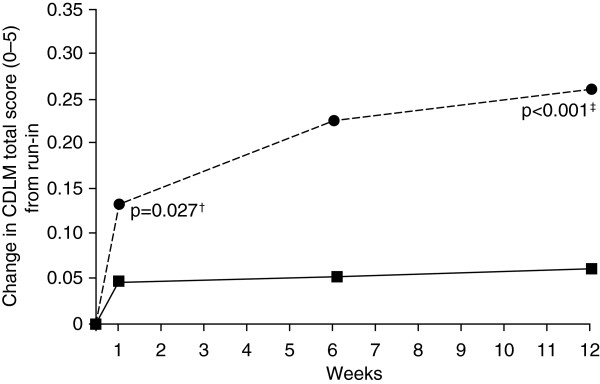
**Change in morning activities (absolute scores) over treatment period with budesonide/formoterol plus tiotropium versus placebo plus tiotropium **[74]**.** CDLM: Capacity of Daily Living during the Morning questionnaire; dotted line represents budesonide/formoterol plus tiotropium; solid line represents placebo plus tiotropium; CDLM score at run-in budesonide/formoterol plus tiotropium: 4.09, placebo plus tiotropium: 4.13; ^†^treatment comparison from randomization to first week of treatment; ^‡^treatment comparison from randomization to last week of treatment. Reprinted with permission of the American Thoracic Society. Copyright © 2013 American Thoracic Society. Welte T, Miravitlles M, Hernandez P, Eriksson G, Peterson S, Polanowski T, et al: Efficacy and tolerability of budesonide/formoterol added to tiotropium in patients withchronic obstructive pulmonary disease. Am J Respir Crit Care Med 2009, 180:741–750. Official journal of the American Thoracic Society.

Compared with a control group, tiotropium amplified the effectiveness of PR in patients with severe and very-severe COPD as demonstrated by increases in patient self-reported participation in physical activities outside the PR program [73]. A recent study investigated the impact of tiotropium added to budesonide/formoterol (combination of inhaled corticosteroid and long-acting beta-2 agonist) in patients with COPD [74]. Significant improvements were observed in the morning activities as assessed by the Capacity of Daily Living during the Morning (CDLM) questionnaire (total score and for individual questions) with tiotropium added to budesonide/formoterol compared with tiotropium alone (Figure 3). This finding is of particular interest in COPD since patients report that morning symptoms are most challenging and heavily impact social and physical activities [75].

Results from a large multi-center trial designed to evaluate the impact of pharmacologic intervention with tiotropium on lung function and the amount of physical activity using an activity monitoring device in patients with GOLD stage II COPD [76] who are not receiving maintenance therapy are awaited [77]. An additional focus of the study will be to assess if efforts to improve physical activity levels early in the course of the disease may contribute to reducing the burden of the morbidity in patients with COPD.

### The way forward

As discussed, the link between bronchodilation, PR, maximal physical performance and physical activity is far from clear-cut. The evidence presented suggests that PR programs may enhance physical activity, but effects are heterogeneous across studies. Longer programs, or the inclusion of targeted behavioral interventions may be needed in order to ensure that patients make significant improvements in physical activity in daily life as exercise training alone is unlikely to alter physical activity behavior.[78]. Nevertheless, studies conducted in patients with mild, moderate and severe COPD have shown a positive impact of PR programs on exercise tolerance and exercise induced symptoms, which could potentially lead patients to become more active in daily life [79-82]. This evidence argues in favor of the adoption of PR in patients with a wide range of COPD severities, including mild COPD, in line with the GOLD 2013 recommendation [2].

Although several studies have assessed the impact of bronchodilators on hyperinflation and exercise endurance capacity in COPD [83-85], evidence on the impact of bronchodilators on physical activity is still scarce. A study in 23 COPD patients evaluating the effect of indacaterol on physical activity measured by an accelerometer over 4 weeks suggested that bronchodilator therapy can improve physical activity [86]. However, when activity level were measured using a sensewear arm band device in a larger study conducted in 90 COPD patients, treatment with indacaterol failed to show any significant improvement versus placebo [87]. The duration of the treatment, differences in the target population and sub-optimal data processing could have played a role in the discrepancies between the two studies. Based on existing evidence, isolated bronchodilator therapy seems unlikely to achieve increases in physical activity, since even PR is often considered to have an inconsistent effect on daily physical activity levels [88-91], unless provided for a prolonged period [78,92]. On a conceptual level, it is possible that bronchodilators could be effective in patients whose main obstacle to physical activity is dyspnea. However, it is also probable that bronchodilators have no effect on physical activity unless combined with some sort of behavioral intervention and/or PR. Preliminary evidence suggests that a counseling program can increase activities of daily living in the absence or presence of a PR program [38,39]. Further investigation to assess the extent to which this should become a standard part of both the physical and pharmacological therapy of COPD is warranted.

It is interesting to note that several interventions that enhance exercise capacity do not seem to have an effect on physical activity. This could be due to several factors including the choice of patient inclusion criteria and tools used to assess physical activity in clinical trials. In designing future studies investigating physical activity in patients with COPD, it may be desirable to stratify participants by physical activity levels measured objectively. As the relationship between GOLD stage and physical activity level is ambiguous [5,9,93], direct measurement of physical activity levels or use of a questionnaire surrogate [33] in future studies will better aid stratification than forced expiratory volume in one second (FEV_1_). In addition, new tools able to capture physical activity from the patient’s perspective are required. Currently available tools measure the amount of physical activity, but they fail to capture the full patient experience of physical activity with regards to symptoms, which have a considerable impact and, to a certain degree, will determine to what extent patients engage in the activities. A comprehensive patient-centered approach combining objective information on the amount of activity with subjective patient experience could be beneficial as it may also help identify the barriers to physical activity in patients with COPD. This may assist in the development of new and comprehensive interventions with the specific aim of increasing physical activity.

## Conclusion

Physical activity is reduced in patients with COPD. This is associated with a higher risk of hospital admission and an increased risk of mortality, and also places patients with COPD at risk of developing comorbidities. Importantly, physical activity is a potentially modifiable risk factor. It follows that improving physical activity allows the patients to take a productive part in daily life and may also confer long term health benefits. The assessment of physical activity and the interpretation of results is an area that has garnered considerable interest. Both subjective and objective instruments for evaluating physical activity have advantages and disadvantages. Patient compliance, appropriate assessment period, and accurate interpretation of data are essential for a precise estimation of daily physical activity. There is limited and inconsistent evidence on the effectiveness of interventions (PR and bronchodilators) for improving physical activity. These inconsistencies may be partly due to patient choice and whether they choose to maintain the life style options explained during their pulmonary rehabilitation. A combination of individualized PR programs and pharmacotherapy in conjunction with behavioral modification may be the way forward to help patients adopt a more active lifestyle.

## Abbreviations

CDLM: Capacity of daily living during the morning; COPD: Chronic obstructive pulmonary disease; FEV1: Forced expiratory volume in one second; GOLD: Global initiative for chronic obstructive lung disease; HRQoL: Health-related quality of life; MLTPAQ: Minnesota leisure time physical activity questionnaire; MLTPAS: Minnesota leisure time physical activity survey; PA: Physical activity; PASE: Physical activity scale for the elderly; PR: Pulmonary rehabilitation; PRO: Patient reported outcomes; QoL: Quality of life; UPLIFT: Understanding potential long-term impacts on lung function with tiotropium; WHO: World health organization; ZPAQ: Zutphen physical activity questionnaire.

## Competing interests

Thierry Troosters received speakers fees from Novartis, Boehringer Ingelheim, Chiesi, AstraZeneca. He is the scientific coordinator of PROactive. Michael Polkey has received fees for speaking or consultancy from GSK, AZ, Novartis and Chiesi. He has attended scientific meetings as a guest of GSK and Almirall. His organization has received on his behalf fees for consultancy from Lilly and GSK and has or holds research grants from AstraZeneca and GSK. Thys van der Molen has received consultancy fees for advisory boards from AstraZeneca, Nicomed, MSD, Novartis, Almirall and speaker fees from AstraZeneca, GSK, Nicomed, Novartis and MSD. His organization has received on his behalf research grants from AstraZeneca, GSK, Nicomed, MSD and Almirall. Roberto Rabinovich, Idelle Weisman, Karoly Kulich and Ioannis Vogiatzis declare that they have no competing interests in relation to this article. All authors are members of the PROactive consortium, IMI JU # 115011.

## Authors’ contributions

All authors were involved in the concept and design of this article. All authors revised the article critically for important intellectual content and gave their final approval of the version to be published.
